# Detection of Alpha-Toxin and Other Virulence Factors in Biofilms of *Staphylococcus aureus* on Polystyrene and a Human Epidermal Model

**DOI:** 10.1371/journal.pone.0145722

**Published:** 2016-01-07

**Authors:** P. M. den Reijer, E. M. Haisma, N. A. Lemmens-den Toom, J. Willemse, R. A. Koning, J. A. A. Demmers, D. H. W. Dekkers, E. Rijkers, A. El Ghalbzouri, P. H. Nibbering, W. van Wamel

**Affiliations:** 1 Department of Medical Microbiology and Infectious Diseases, Erasmus University Medical Center, Rotterdam, The Netherlands; 2 Department of Infectious Diseases, Leiden University Medical Center, Leiden, The Netherlands; 3 Department of Dermatology, Leiden University Medical Center, Leiden, The Netherlands; 4 Department of Molecular Cell Biology, Leiden University Medical Center, Leiden, The Netherlands; 5 Proteomics Centre, Erasmus University Medical Center, Rotterdam, The Netherlands; LSU Health Sciences Center School of Dentistry, UNITED STATES

## Abstract

**Background & Aim:**

The ability of *Staphylococcus aureus* to successfully colonize (a)biotic surfaces may be explained by biofilm formation and the actions of virulence factors. The aim of the present study was to establish the presence of 52 proteins, including virulence factors such as alpha-toxin, during biofilm formation of five different (methicillin resistant) *S*. *aureus* strains on Leiden human epidermal models (LEMs) and polystyrene surfaces (PS) using a competitive Luminex-based assay.

**Results:**

All five *S*. *aureus* strains formed biofilms on PS, whereas only three out of five strains formed biofilms on LEMs. Out of the 52 tested proteins, six functionally diverse proteins (ClfB, glucosaminidase, IsdA, IsaA, SACOL0688 and nuclease) were detected in biofilms of all strains on both PS and LEMs. At the same time, four toxins (alpha-toxin, gamma-hemolysin B and leukocidins D and E), two immune modulators (formyl peptide receptor-like inhibitory protein and Staphylococcal superantigen-like protein 1), and two other proteins (lipase and LytM) were detectable in biofilms by all five *S*. *aureus* strains on LEMs, but not on PS. In contrast, fibronectin-binding protein B (FnbpB) was detectable in biofilms by all *S*. *aureus* biofilms on PS, but not on LEMs. These data were largely confirmed by the results from proteomic and transcriptomic analyses and in case of alpha-toxin additionally by GFP-reporter technology.

**Conclusion:**

Functionally diverse virulence factors of (methicillin-resistant) *S*. *aureus* are present during biofilm formation on LEMs and PS. These results could aid in identifying novel targets for future treatment strategies against biofilm-associated infections.

## Introduction

*Staphylococcus aureus* (*S*. *aureus*) is the causative agent of a variety of infections with generally significant morbidity and mortality. The incidence of both hospital and community acquired infections caused by methicillin-resistant *S*. *aureus* (MRSA) has increased significantly in the last decades [1–3]. Unfortunately, the treatment of such infections is becoming increasingly complex as current antibiotics may be less effective due to resistance development and biofilm formation [4]. As the number of newly approved antimicrobial agents continues to decrease [5,6], alternative strategies for prevention and/or treatment of bacterial colonization and infection, such as a vaccines [7] and antimicrobial peptides [8], are urgently needed. To date no clinically successful vaccine against *S*. *aureus* has been developed, despite the promising results of vaccines targeting diverse virulence factors of this pathogen in animal models [9,10]. Currently, the awareness that multiple virulence factors of *S*. *aureus* should be targeted for any vaccine or other strategy to be successful is increasing [9]. Moreover, some relation between the expression of antibodies against *S*. *aureus* virulence factors and protection from infection has been made [11].

The capacity of *S*. *aureus* to cause infections is attributed to its vast array of virulence factors which include adhesive surface proteins, secreted immune modulators, enzymes and toxins [7]. Moreover, many infections such as those of (wounded) skin, mucosae and artificial surfaces [12] are believed to involve biofilm formation by *S*. *aureus*. Biofilms are defined as complex communities of bacteria encased in an extracellular polymeric matrix and biofilm formation is believed to contribute to bacterial virulence, reduced susceptibility to antibiotics [13–15] and reduced clearance by the immune system. Despite the plethora of studies examining the involvement of biofilm formation [16] and/or single virulence factors [17,18] in e.g. skin infections, so far only a few studies has focussed on the involvement of multiple virulence factors in association with biofilm formation by *S*. *aureus* during infection [19,20].

Biofilm formation by *S*. *aureus* on polystyrene (PS) has been extensively characterized before [21,22]. However, biofilm formation on human biotic surfaces is much less characterized and the associated pathogen-host interactions are unclear. Earlier we reported that Leiden epidermal models (LEMs) mimic the human skin in many ways, including epidermal morphology and barrier properties [23]. In addition, full thickness human skin equivalents have been used to study skin colonization by (methicillin resistant) *S*. *aureu*s [24,25].

The aim of the current study was to establish the presence of 52 proteins, including virulence factors such as alpha-toxin, during biofilm formation by five different (methicillin-resistant) *S*. *aureus* strains on LEMs and PS. Using the novel competitive Luminex-based assay (CLA; [26] we detected six proteins (ClfB, glucosaminidase, IsdA, IsaA, SACOL0688 and nuclease) in biofilms of all biofilm-forming strains on the two surfaces. At the same time, surface- and strain-dependent differences were found for the presence of a wide range of other proteins, such as immune modulators and toxins like alpha-toxin.

## Materials and Methods

### Ethics statement

Human serum was obtained from healthy volunteers who gave written consent for use of serum solely for research purposes within the department of Medical Microbiology and Infectious Diseases at the Erasmus MC Rotterdam. Serum was coded, pooled and has been used for this and earlier studies [27,28]. The original list with documented volunteer names was only accessible to qualified physicians within the department, amongst the current authors only including PMdR. This sampling procedure was approved by the Medical Ethics Committee of the Erasmus Medical Center Rotterdam (MEC-2007-106, addendum 2) [28]. All primary human skin cells from healthy donors used by the Department of Dermatology are isolated from surplus tissue collected according to article 467 of the Dutch Law on Medical Treatment Agreement and the Code for proper Use of Human Tissue of the Dutch Federation of Biomedical Scientific Societies [29]. According to article 467, coded anonymous surplus tissue can be used if no objection is made by the patient. All patients were informed of the possibility that surplus tissue could be used for scientific research and all patients were offered the opportunity to give written refusal to this. Only tissue from patients who did not opt out was used. None of the authors were involved in the tissue sampling and only birth date, gender and skin type of the subjects were documented. These data were only accessible to EMH and PHN. Because this procedure, as published previously [8,24–25], is in accordance with national law and additional approval of an ethics committee regarding scientific use of surplus tissue is not required, we did not seek specific approval by our ethics committee. The Declaration of Helsinki principles were followed when working with human tissue.

### *Staphylococcus aureus* strains

The following *S*. *aureus* strains were used in this study: methicillin-resistant strains LUH14616 (sequence type 247), a kind gift of dr. S. Croes [30]; LUH15051 (ST 239) obtained from dr. M.E.O.C. Heck, (Laboratory of Infectious Diseases and Screening, RIVM, Bilthoven, The Netherlands); USA300 strain Sac042w (ST 8) described earlier [31]; a strain derived from an impetigo patient LUH15091 (ST121) within the Erasmus Medical Center and NCTC 8325–4 (ST 8). All strains were typed using multi locus sequence typing (MLST) [27,32]. Before usage the strains were grown on sheep blood agar plates (Biomerieux).

### Biofilm formation on polystyrene plates

A routine biofilm model was used as described before [21,22]. In short, overnight plate cultures of *S*. *aureus* were re-suspended in Iscove’s Modified Dulbecco’s Medium (IMDM, Life technologies, Carlsbad, CA, USA) without phenol red until an optical density (OD, 660nm) of 2 was reached. IMDM medium was chosen because of its significant impact on detectable levels of bacterial proteins, e.g. IsdA, ClfB and Efb, expressed by *S*. *aureus* biofilms on PS [26]. One μl of this bacterial suspension was added to 199 μl of TSB supplemented with 0.5% (wt/v) glucose and 3% (wt/v) NaCl or IMDM without any supplement in sterile 96-wells PS plates (Greiner Bio-one). Plates were then incubated at 37°C with gentle shaking at 200 rpm for various intervals. Biofilm mass was measured by staining with 1% crystal violet. OD was measured at 490 nm.

### Leiden epidermal models

**Fig 1:**
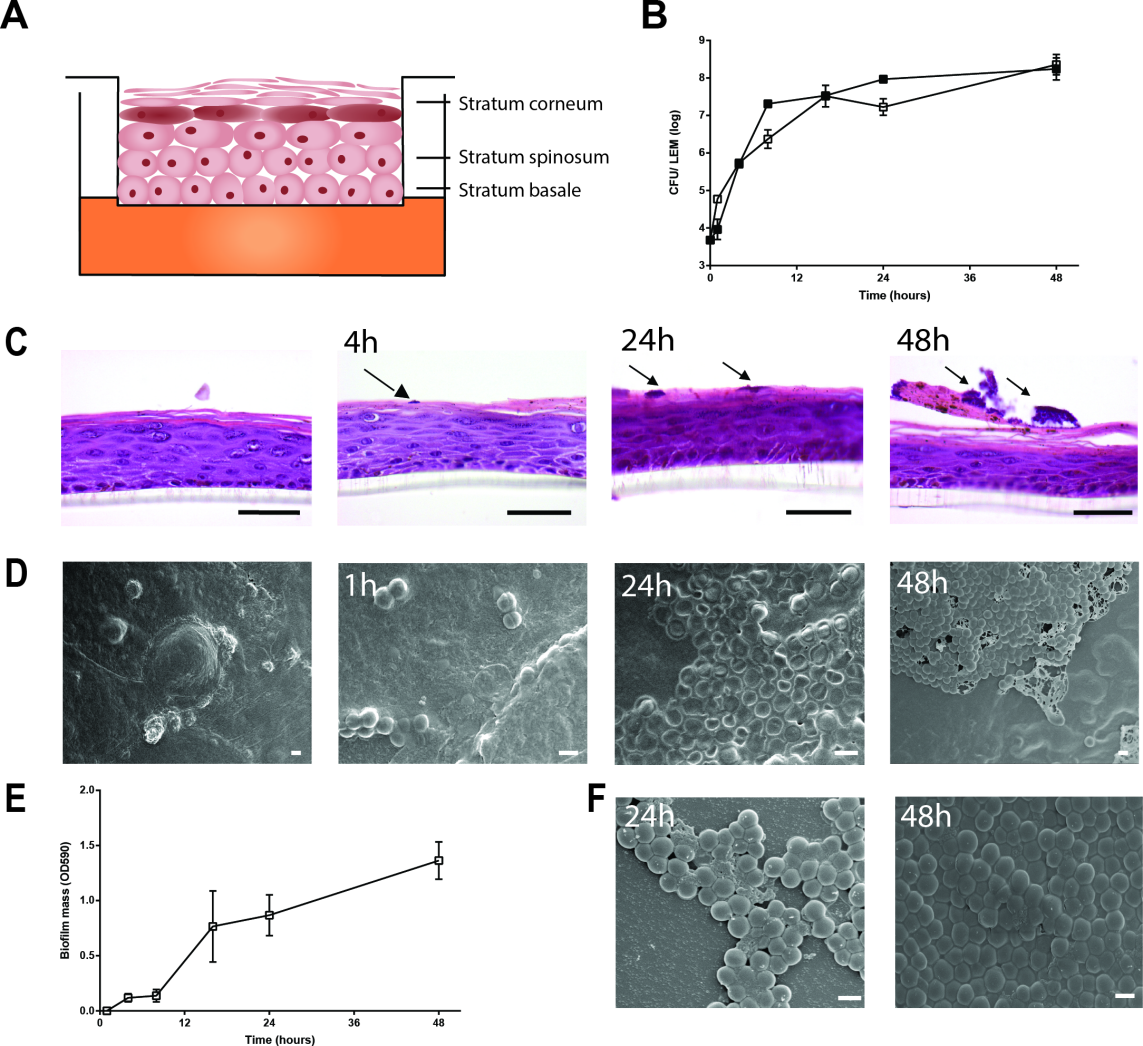
Biofilm formation by *S*. *aureus* LUH14616 on LEMs and PS surfaces. (**A**) Schematic representation of LEM. (**B**) Bacterial counts were performed on LEM exposed to LUH14616 for various intervals. Adherent bacteria are represented by open symbols and non-adherent/loosely adherent bacteria by closed symbols. Results are displayed as the mean and SD of four experiments. (**C**) Haematoxilin and eosine staining of LEMs at various intervals after inoculation with LUH14616. Arrows indicate microcolonies, scale bars = 50 μm. (**D**) Cryo scanning electron microscopy of LEMs colonized with LUH14616 for various intervals. Photographs are representative for three different keratinocyte donors. (**E**) Biofilm formation by LUH14616 on PS in IMDM medium. Results are the mean and SEM of three experiments. (**F**) Cryo scanning electron microscopy of *S*. *aureus* LUH14616 biofilms formed on PS at 24 and 48 hrs after adherence to wells. Scale bars = 1 μm.

The epidermis and dermis of pieces of fresh plastic surgery surplus skin were enzymatically and mechanically separated, and each layer was subsequently digested to obtain single-cell suspensions [33]. The keratinocytes were cultured in keratinocyte medium, i.e. 3 parts DMEM (Gibco/Invitrogen) and 1 part Ham's F12 medium supplemented with 5% (v/v) fetal bovine serum (FBS; HyClone/Greiner), 0.5 μm hydrocortisone, 1 μm isoproterenol, 0.1 μm insulin (all from Sigma–Aldrich, Zwijndrecht, The Netherlands), 100 U/ml penicillin and 100 μg/ml streptomycin (both from Invitrogen). Leiden epidermal models (LEMs; Fig 1A) were made with these primary human keratinocytes as described before [34]. Briefly, one day before generation of the models, medium of the keratinocyte cultures was switched to Dermalife (Lifeline Cell Technology) supplemented with penicillin (10,000 U/ml) and streptomycin (10 mg/ml). The next day 2×10^5^ keratinocytes were seeded onto a filter insert (0.4 μM Costar inserts; Corning) in 12-well plates in this Dermalife medium. Three days thereafter the apical medium was removed, leaving the keratinocytes air-exposed. The medium under the filter insert was switched to CnT-02-3D medium (CellnTech) mixed with keratinocyte medium supplemented with 2.4×10^−2^ μM bovine serum albumin and lipids/antibiotics as described above. One day before bacterial inoculation of the LEMs, the medium was switched to this medium without antibiotics. All experiments were performed on 10-day air-exposed cultures.

### Colonization of Leiden epidermal model

LEMs were exposed for 1 h at 37°C in 7.3% CO_2_ to 300 μl of a log-phase bacterial suspension containing 3.3x10^5^ CFU/ml. Next, the non-adherent bacteria were removed by aspiration and at different intervals thereafter, the viable detachable bacteria were collected in 1 ml of PBS, and serially diluted, and 50 μl of these samples were plated onto diagnostic sensitivity test (DST) agar plates (Oxoid) to determine the number of CFU. To assess the number of adherent bacteria, a model was cut in two equally sized pieces. One piece was used for histology and the other was homogenized in PBS using a glass Potter-Elvehjem tissue homogenizer, and the homogenates were subsequently serially diluted and plated as described above. The lower limits of detection for detachable and adherent bacteria were 20 and 40 CFU/LEM, respectively.

### Histology

One biopsy of each model was fixed in 4% (v/v) formaldehyde, dehydrated, and embedded in paraffin. Next, paraffin blocks were cut into 5-μm sections, deparaffinized, rehydrated, and then stained with hematoxylin and eosin (H&E) staining.

### Cryo scanning electron microscopy

For the morphological study of (methicillin resistant) *S*. *aureus* biofilms on LEM or PS by cryo-scanning electron microscopy (SEM), specimens were quickly frozen in liquid nitrogen slush and transferred directly to the cryo-transfer attachment (Gatan Alto2500). Samples were sublimated at -90 in high vacuum for 5 min and subsequently sputter-coated with a layer of 20 nm gold/paladium and examined in a JEOL JSM6700F scanning electron microscope.

### Multiplex bead assay for assessment of the presence of proteins during *S*. *aureus* biofilm formation

A multiplex competitive Luminex assay [26] (CLA) with minor modifications was used to indirectly detect the presence of 52 IgG-accessible proteins in bacterial cultures (all bacterial proteins are described in S1 File). In brief, log-phase cultures of *S*. *aureus* were diluted 1:200 and incubated in PS wells for 1, 8, 24 and 48 hrs. After washing with ice-cold PBS supplemented with 0.5% (wt/v) sodium azide (Sigma-Aldrich), adherent bacteria residing in biofilms on PS or LEMs were incubated at 8°C and continuous shaking (500 rpm) with 200 μl of a 1:200 dilution of polyclonal human IgG (PHG), isolated using the HiTrap™ Protein G HP column according to the manufacturer’s guidelines (GE Healthcare Bio-sciences, Piscataway, New Jersey, USA), from pooled serum of 40 healthy volunteers (19 non-nasal carriers, 6 intermittent and 15 persistent nasal carriers of *S*. *aureus* as determined earlier [35]. After 35 min incubation the PHG samples were recovered from biofilms. The remaining non-bound IgG antibody levels in these samples, specifically directed against 52 proteins of *S*. *aureus*, were measured using a multiplex bead-based flow cytometry technique (xMAP®, Luminex corporation) wherein recombinant proteins were covalently coupled to the beads as described previously [27,28,36]. As negative controls PHG samples incubated with empty PS wells or sterile LEMs were included in all experiments.

Next, the percentage decrease in the levels of specific IgG antibodies for each protein was calculated in relation to the negative control. The percentage decrease can be considered a semi-quantitative measure of the protein-specific antibody absorption from PHG by the biofilm, thus indirectly reflecting the presence of the particular *S*. *aureus* protein by the biofilm [26]. The average percentage decrease plus two times the standard deviation, obtained at 8, 24 and 48 hours biofilm growth, for the three non-*S*. *aureus* control proteins and all *S*. *aureus* proteins of which genes were not present in LUH14616 were chosen as cut-off value (35% at 8 and 24 hrs biofilm growth and 40% at 48 hrs, respectively).

In case of bacteria adherent to LEMs, the same protocol was followed with the single modification that PHG samples were directly incubated on top of the LEM. To determine the presence of *S*. *aureus* proteins in culture supernatants, growth medium that covered biofilms grown on PS was analyzed using the same protocol, with the modification that medium was removed at designated time points and incubated with PHG samples in sterile wells.

### Reverse transcriptase PCR

Early biofilms (8 and 16 hrs) were grown in 96-well plates (Cellstar culture plates, Greiner Bio-One) in 200 μl of IMDM (Gibco). Biofilms were resuspended, pooled and centrifuged at 4000 rpm for 10 min at 4°C. Pellets were resuspended in 200 μl of RNA protect™ Bacterial reagents (Qiagen), stabilized for 5 min and then centrifuged for 10 min at 4°C. The pellet was dissolved in 1 ml of RNA-pro solution (Fast RNA Pro Blue kit, MP Biomedicals) and stored at -20°C until use. RNA was isolated using the Fast RNA Pro Blue kit according to the manufacturer’s protocol. Each 10 μg of isolated RNA was treated twice with 2 U TURBO DNase (Ambion, Life Technologies). The reaction was stopped by adding 0.2 volumes of DNase inactivation reagent (Ambion) and incubation for 2 min at ambient temperature. RNA containing supernatants were collected by centrifugation (1.5 min at 9000 g at ambient temperature) and each 2 μg DNase-treated RNA was treated with 2 U DNase I (Fermentas, Fisher Scientific). One μg of prepared RNA was transcribed into cDNA using 200 U RevertAid H Minus Reverse transcriptase (Fermentas), 4 μl of 5x reaction buffer (Fermentas), 20 U of RiboLock RNase inhibitor (Fermentas) and 2 μl of 10 mM dNTP mix (Fermentas) in a final volume of 20 μl of DEPC-treated water. This was incubated for 60 min at 42°C and then terminated by heating at 70°C for 5 min. For each RNA sample a negative control without reverse transcriptase was processed similarly. The presence of cDNA in all samples was examined using PCR as described previously [27].

### Proteomics

A total of 48 biofilms of strain LUH14616 were grown for 8 hours in PS wells as described above, resuspended in 200 μl of PBS per well, pooled and spun down at 4,000 rpm for 5 min. The resulting pellet was resuspended in 50 μl of PBS, mixed with 50 μl of Laemni buffer and heated for 5 min at 95°C. Fifty μl of this suspension containing denatured proteins were run on a 15% SDS gel (Biorad) and gel lanes were cut into ~1 mm slices. Lanes were subjected to in-gel reduction with dithiothreitol, alkylation with chloroacetamide and digestion with trypsin (Promega, Leiden, The Netherlands). Nanoflow LC-MS/MS was performed on an 1100 series capillary LC system (Agilent Technologies) coupled to an LTQ Orbitrap XL mass spectrometer (Thermo), operating in positive mode and equipped with a nanospray source. Mass spectra were acquired in continuum mode; fragmentation of the peptides was performed in data-dependent mode by CID. Peak lists were automatically created from raw data files using the Proteome Discoverer (version 1.3; Thermo). The Mascot search algorithm (version 2.2, MatrixScience) was used for searching against the Uniprot database (release 2013_06.fasta, taxonomy: *S*. *aureus*, strains USA300, Newman, NCTC 8325–4 and COL). The peptide tolerance was set to 10 ppm and the fragment ion tolerance was set to 0.8 Da. A maximum number of 2 missed cleavages by trypsin were allowed and carbamido-methylated cysteine and oxidized methionine were set as fixed and variable modifications, respectively. The Mascot score cut-off value for a positive protein hit was set to 65. Individual peptide MS/MS spectra with Mascot scores below 25 were checked manually and either interpreted as valid identifications or discarded.

### Construction of the *hla* promotor upstream of GFP_uvr_

*S*. *aureus* strains LUH14616 and Sac042w containing a vector with an *hla* promotor upstream of GFP_uvr_ were prepared as described earlier [37] with some modifications. First, the promotor of *hla* was amplified using primers hlapr1 (cggaattcgatatttctatgtaatggca) and hlapr2 (gctctagacttctatttttttgaacgat) and as a template DNA from *S*. *aureus* strain Newman. Next, the amplification product was ligated into the EcoRI XbaII site of pALC1484 (a kind gift from dr A.L. Cheung, Dartmouth College, New Hampshire, US) and cloned into *E*. *coli* DH10beta. From positive colonies on LB agar supplemented with 50 μg of ampicillin/ml, recombinant plasmids were isolated, checked by PCR and sequencing, and then electroporated into *S*. *aureus* RN4220. Finally, from positive colonies on BHI agar supplemented with 10 μg of chloramphenicol/ml, plasmids were isolated and electroporated into *S*. *aureus* LUH14616 and Sac042w. As positive and negative control we electroporated respectively pWVW 163, a plasmid containing a phage promotor yielding a strong, constant expression of GFP, and pALC1484, an empty vector [38], into the same *S*. *aureus* strains as described above.

### Data analysis

All data were analysed using Microsoft Excel version 2010 and graphics were made using Graphpad Prism version 5 (Graphpad Inc. La Jolla, CA, USA).

## Results

### Biofilm formation by MRSA strain LUH14616 on LEMs and PS

Firstly, the ability of the clinical isolate MRSA LUH14616 to form biofilms on both the human skin model (Leiden Epidermal Model: LEM, schematically represented in Fig 1A), and polystyrene (PS) was examined. This MRSA strain was able to adhere to and stably colonize both surfaces, as reflected by an increase in bacterial counts on LEM (Fig 1B) and an increase in crystal violet staining on PS (Fig 1E) within the first 24 hrs after inoculation. Interestingly, haematoxylin-eosin staining of the colonized LEMs showed that the bacteria adhered to the *stratum corneum* and formed small colonies after 16 hrs, but did not invade the epidermis (Fig 1C). To further examine biofilm formation by MRSA strain LUH14616 on these surfaces, bacterial colonization on LEM (Fig 1D) and PS (Fig 1F) was visualized with scanning electron microscopy. Results revealed a tightly adherent layer of bacteria on both LEMs and PS after 24–48 hrs, indicating the development of a mature biofilm on both surfaces. Biofilm-associated bacteria on LEM appeared to be completely encased in an extracellular matrix (Fig 1D), while bacteria on PS appeared to be incompletely encased (Fig 1F).

### Detection of toxins, immune modulators and other proteins of MRSA strain LUH14616 during biofilm formation on PS

We used a competitive Luminex-based assay (CLA) to establish the presence of 52 bacterial proteins during biofilm formation by MRSA strain LUH14616 on polystyrene (PS).

**Fig 2:**
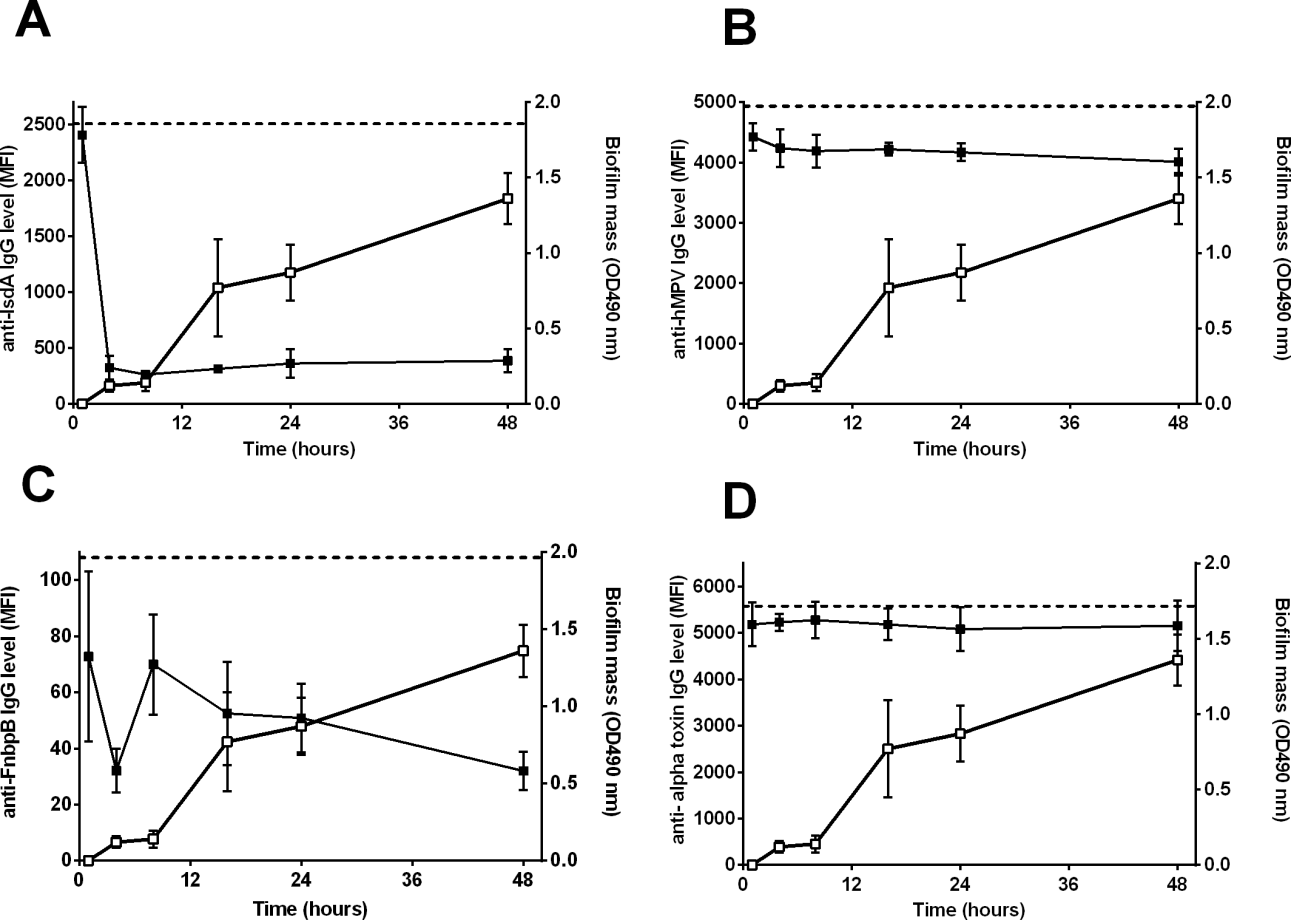
Detection of *S*. *aureus* proteins during biofilm formation of LUH14616 on PS. Closed symbols indicate the mean fluorescence intensity (MFI, left Y-axis), reflecting the level of remaining non-bound IgG directed against specific proteins after incubation of PHG with the biofilms, while open symbols indicate biofilm mass (OD490 nm, right Y-axis). Both are plotted against the time of biofilm growth (hrs). Results are shown for **(A)** IsdA, **(B)** control protein of human metapneumovirus (hMPV)**, (C)** FnbpB and (**D**) alpha toxin. Dashed horizontal lines indicate the average MFI of sterile controls. Symbols and error bars indicate mean and SD of four experiments.

In line with previous results [26], biofilm mass-dependent absorption of specific IgG for several *S aureus* proteins, such as IsdA (Fig 2A), FnbpB (Fig 2C) and glucosaminidase by biofilms was detected, while no such reduction was seen for the levels of IgG antibodies directed against control proteins, e.g. the protein derived from human metapneumovirus (hMPV) (Fig 2B). Based on the percentage decrease in the levels of IgG directed against the three non-*S*. *aureus* control proteins and against the 28 *S*. *aureus* proteins of which genes were not found in LUH14616 using PCR, cut-off values of at least 35% decrease in specific IgG at 24 hrs biofilm growth and 40% at 48 hrs were calculated. CLA measurements for five proteins [ESX-1-associated factors EsxA and EsxB, iron surface determinants H (IsdH), Staphylococcal enterotoxin J (SEJ) and foldase-protein PrsA] were excluded from further analysis due to low MFI’s with standard deviations larger than 25% between repeated CLA measurements.

Using the above mentioned cut-off values, we detected 8 proteins in 24 hrs and 48 hrs-old *S*. *aureus* biofilms: the surface proteins fibronectin-binding protein B (FnbpB), CflB, glucosaminidase, iron-responsive surface determinant A (IsdA), immunodominant antigen A (IsaA), SACOL0688, nuclease, and the immune modulator Efb (Table 1). In addition, a significant decrease in the levels of IgG specifically directed against chemotaxis inhibitory protein of *S*. *aureus* (CHIPS) with 48 hrs-old biofilms, but not 24 hrs-old biofilms, was observed. No significant decrease in the levels of specific IgG for 15 other proteins, despite the presence of corresponding genes in LUH14616 such as for alpha toxin, was observed (Fig 2D). Additional experiments showed that the secreted proteins alpha-toxin, HlgB, FLIPr and SSL1 could neither be detected in the growth medium covering biofilms, excluding the possibility of false-negative signals for these secreted proteins (S1 Fig). CLA data were further validated by confirming the presence of 7 out of the 8 detected proteins in early (8 hrs) biofilms using mass-spectrometry, while mRNA was detected for 5 of these proteins in early biofilms.

**Table 1:** Detection of mRNA and proteins during biofilm formation of LUH14616 on LEMs and PS.

			Biofilms on polystyrene		Biofilms on polystyrene			Biofilms on LEM	
			mRNA present^3^	Protein detectable^4^	Significant reduction in specific IgG^5^			Significant reduction in specific IgG^5^	
Protein^1^	Gene^2^	Functional class	8 hrs	8 hrs	8 hrs	24 hrs	48 hrs	24 hrs	48 hrs
CHIPS	*chp*	immmune modulator	Yes	Yes	-	-	+	-	+
ClfB	*clfB*	surface protein	Yes	Yes	+	+	+	+	+
Glucosaminidase	*Atl*	housekeeping	No	Yes	+	+	+	+	+
IsaA	*isaA*	housekeeping	Yes	Yes	+	+	+	+	+
IsdA	*isdA*	surface protein	Yes	Yes	+	+	+	+	+
Nuc	*nuc*	housekeeping/ toxin	No	Yes	+	+	+	+	+
SACOL0688	*MntC*	housekeeping	Yes	Yes	+	+	+	+	+
	* *								
Efb	*efb*	immmune modulator	No	Yes	+	+	+	-	-
FnBPB	*fnbB*	surface protein	Yes	No	+	+	+	-	-
									
Alpha toxin	*hla*	toxin	Yes	No	-	-	-	+	+
FlipR	*flr*	immmune modulator	Yes	No	-	-	-	+	+
HlgB	*hlgB*	toxin	Yes	No	-	-	-	+	+
Lipase	*lip*	housekeeping/ toxin	Yes	No	-	-	-	+	+
LukD	*lukD*	toxin	Yes	No	-	-	-	+	+
LukE	*lukE*	toxin	Yes	No	-	-	-	+	+
LytM	*lytM*	housekeeping	Yes	No	-	-	-	+	+
SSL1	*ssl1*	immmune modulator	Yes	No	-	-	-	-	+
	* *								
FnBPA	*fnbA*	surface protein	No	Yes	-	-	-	-	-
SCIN	*scn*	immmune modulator	Yes	No	-	-	-	-	-
SdrD	*sdrD*	surface protein	Yes	Yes	-	-	-	-	-
SEA	*sea*	toxin	Yes	No	-	-	-	-	-
SSL3	*ssl3*	immmune modulator	Yes	No	-	-	-	-	-
SSL5	*ssl5*	immmune modulator	Yes	No	-	-	-	-	-
SSL10	*ssl10*	immmune modulator	Yes	No	-	-	-	-	-

^1^Only proteins are shown for which corresponding genes were detected in LUH14616 and for which standard deviation between 4 CLA experiments did not exceed 25%. From top to bottom protein groups are shown that were detected on both LEMs and PS, only on PS, only on LEMs or on neither surface, respectively.

^2^Additional ORF IDs for all genes, based on sequences of *S*. *aureus* strain 8325–4 (SAOUHSC) or Newman (NWMN), are available online (http://www.uniprot.org/).

^3^Presence of mRNA was established using RT-PCR in early 8 hrs biofilms on PS.

^4^Presence of proteins was established using mass spectrometry in early 8 hrs biofilms on PS

^5^Significant reduction in the levels of IgG specific for each protein, indicative of the presence of the protein during biofilm formation, was defined as a reduction in IgG (compared to sterile controls) of at least 35% at 8 and 24 hrs biofilm growth and 40% at 48 hrs.

### Detection of toxins, immune modulators and other proteins of MRSA strain LUH14616 during biofilm formation on LEMs

**Fig 3:**
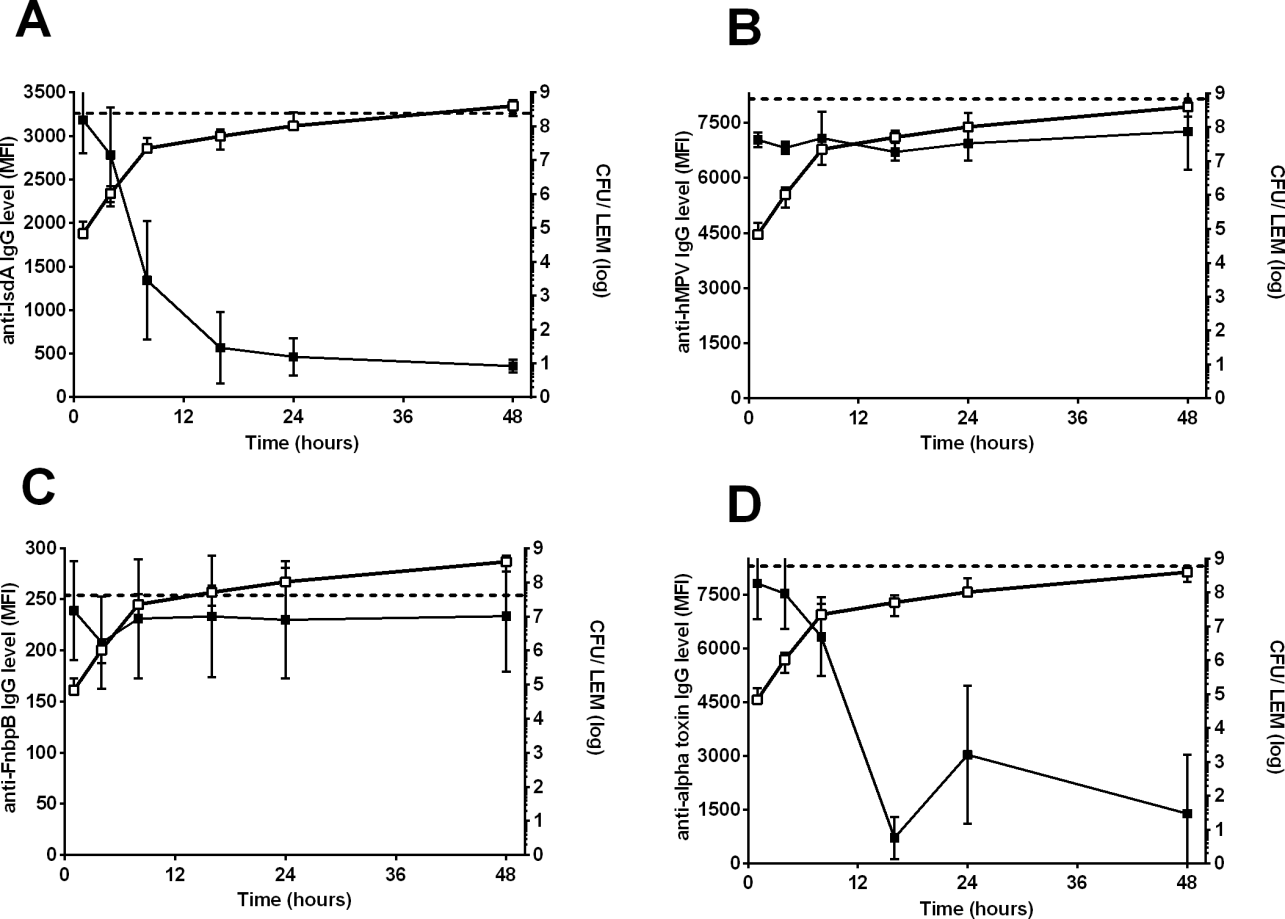
Detection of *S*. *aureus* proteins during biofilm formation of LUH14616 on LEMs. Closed symbols indicate the mean fluorescence intensity (MFI, left Y-axis), reflecting the level of remaining non-bound IgG directed against specific proteins after incubation of PHG with the bacterial biofilms, while open symbols indicate biofilm mass (OD490 nm, right Y-axis). Both are plotted against the time of biofilm growth (hrs). Results are shown for (**A**) IsdA, (**B**) control protein of human metapneumovirus (hMPV), **(C)** FnbpB and(**D**) alpha toxin. Dashed horizontal lines indicate the average MFI of sterile controls. Symbols and error bars indicate mean and SD of four experiments, respectively.

Next we screened for the presence of the same 52 proteins in biofilms of LUH14616 grown on LEM. Similar to biofilms grown on PS we observed time dependent absorption of antibodies against diverse antigens such as IsdA (Fig 3A) and glucosaminidase, whereas no such reduction was observed for antibodies directed against the non-*S*. *aureus* control proteins (Fig 3B) and the proteins of which the gene was not found in LUH14616. This prompted us to use the same cut-off values.

Thirteen proteins were detected in 24 and 48 hrs-old biofilms on LEMs (Table 1): the surface proteins clumping factor B (CflB), glucosaminidase, IsdA, IsaA, glycyl-glysine endopeptidase (LytM), and SACOL0688; the toxins alpha-toxin (Fig 3D), gamma-hemolysin B (HlgB), leukocidins (Luk) D and E, lipase and nuclease; and the immune modulator formyl peptide receptor-like inhibitory protein (FLIPr). In addition, CHIPS and staphylococcal superantigen-like protein 1 (SSL 1) were detected in 48 hrs biofilms, but not 24 hrs biofilms. In contrast to biofilms on PS, no significant reduction was observed for antibodies against FnbpB at any time point (Fig 3C).

### Detection of proteins during biofilm formation on LEMs and PS by different *S*. *aureus* strains

**Fig 4:**
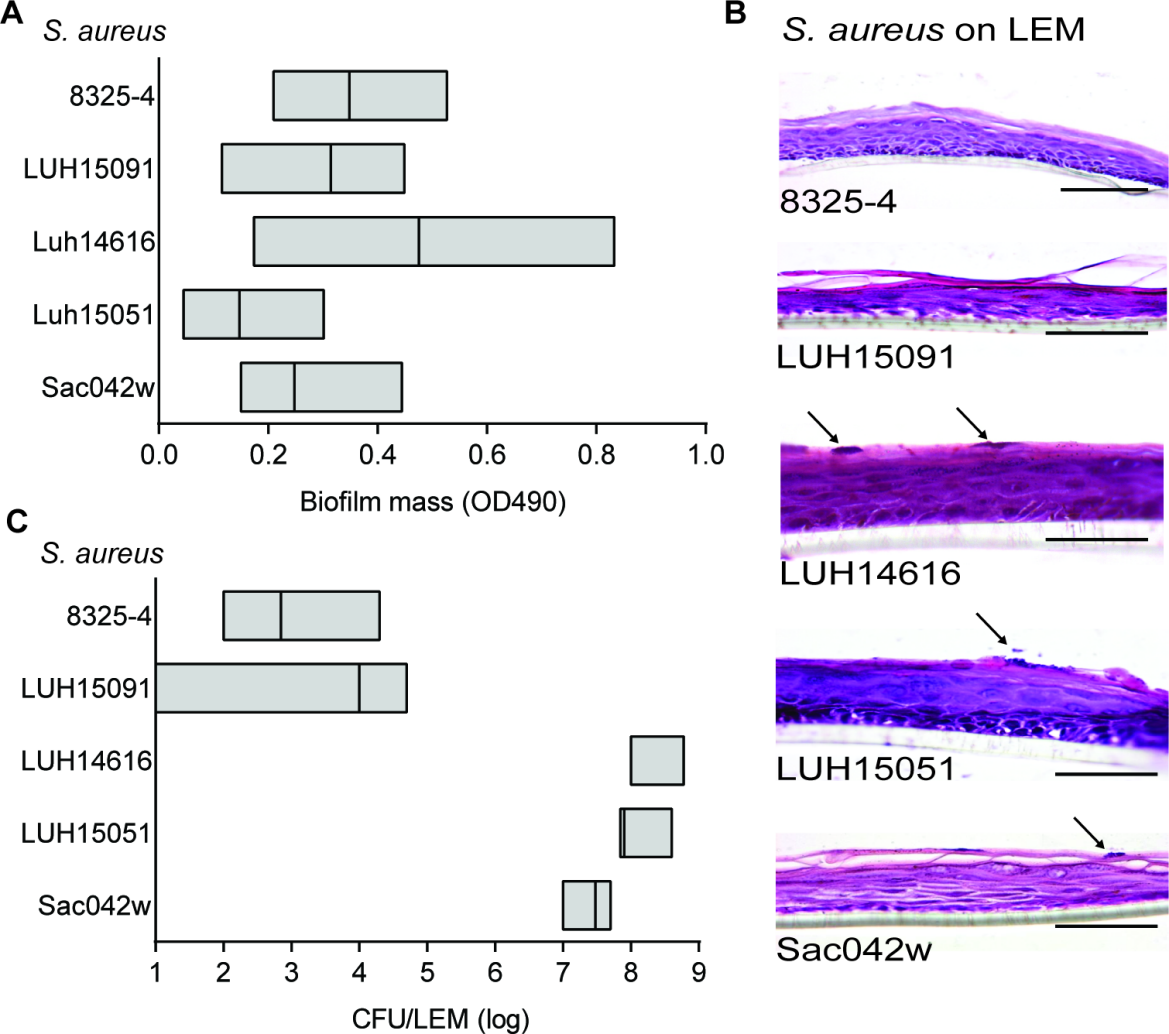
Biofilm formation by 5 different *S*. *aureus* strains on LEMs and PS surfaces. (**A**) Biofilm formation after 24 hrs on PS was measured by crystal violet staining. (**B**) Hematoxylin and eosine staining of LEMs 24 hrs after exposure to *S*. *aureus* 8325–4, LUH15091, LUH15051, LUH14616 or Sac042w, arrows indicate *S*. *aureus*. Photographs are representative for three different experiments. Scale bars = 50 μm. (**C**) The number of viable bacteria present on epidermal models after 24 hrs inoculation was determined microbiologically (CFU/LEM). Results are boxplots showing the median and range. Results are means and SEM of three to five experiments.

To determine whether the results obtained for MRSA LUH14616 are representative for other *S*. *aureus* strains, experiments with 24 hrs-old biofilms of an additional set of four, genetically diverse S. aureus strains were performed: i.e. LUH15051, LUH15091, the USA300 strain Sac042w, and NCTC 8325–4. Results revealed considerable variance in biofilm mass formed on the PS plates and LEMs among the different strains (Fig 4A–4C). Interestingly, strains 8325–4 and LUH15091 formed a significant biofilm on PS, but not on LEMs. The latter two strains were therefore excluded from further analyses. The same cut-off values were used as for LUH14616.

In agreement with the results for LUH14616, the proteins ClfB, IsdA, IsaA, SACOL0688 and glucosaminidase were detected in 24 hrs-old biofilms of LUH15051 and Sac042w on both PS and LEMs (S1 Table). In addition, the toxins HIgB, LukD and E and the immune modulator SSL1 were detected in biofilms of both strains only on LEMs, while FnBPB was detected only on PS. In contrast to results obtained with LUH14616, we additionally detected the proteins CHIPS, efb, lipase and lytM (in biofilms of both LUH15051 and Sac042w) and alpha-toxin and FLipR (Sac042w only) on both surfaces. Finally, we detected SEA in biofilms of LUH15051 and Sac042w on respectively LEMS and PS, while SdrD was detected for both strains on LEMS.

### Alpha-toxin expression by MRSA strains LUH14616 and Sac042w during biofilm formation on LEMs and PS

**Fig 5:**
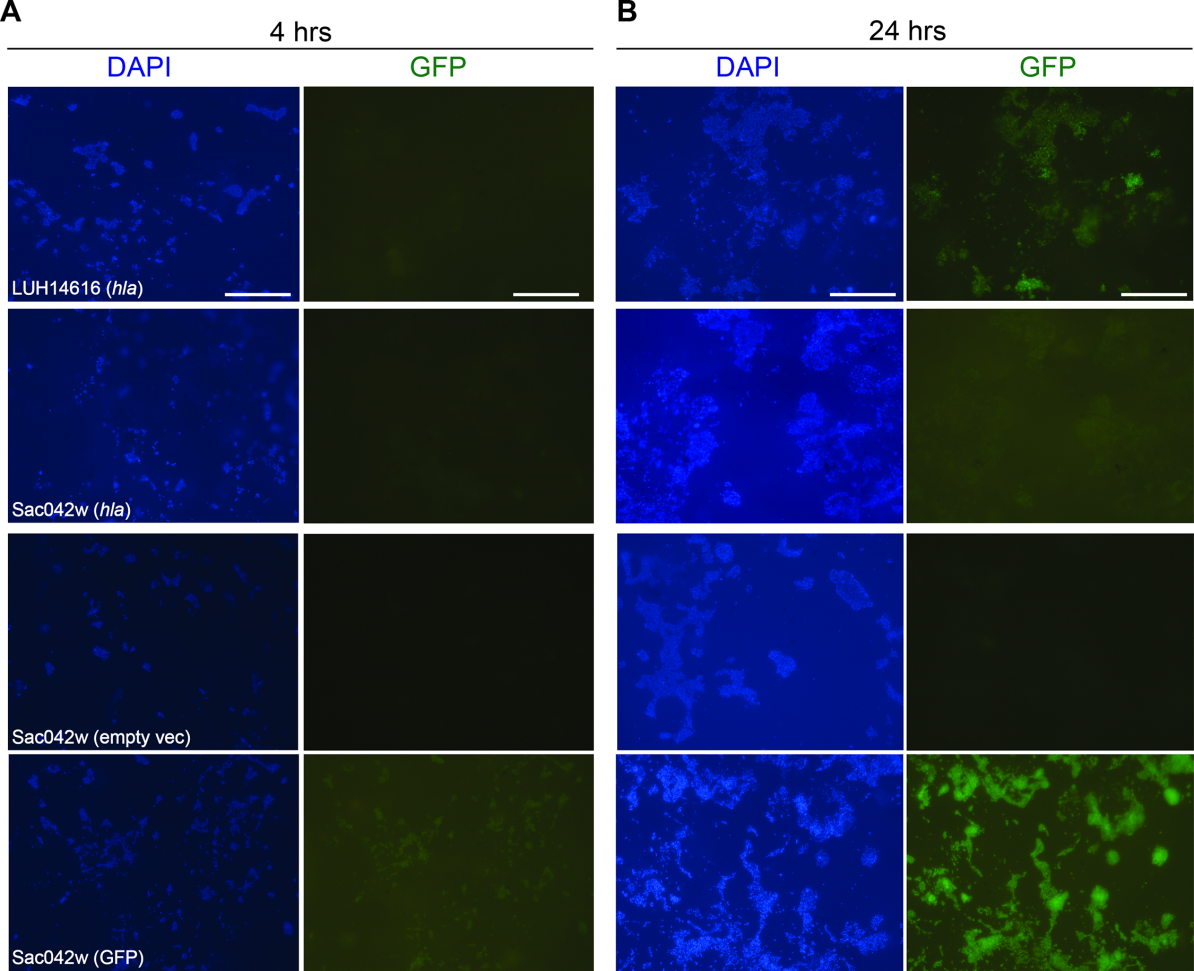
Expression of *hla* by *S*. *aureus* LUH14616 and Sac042w during biofilm formation on LEMs. LUH14616 and Sac042w containing *hla*-GFP (*hla)*, empty vector (empty vec) or a construct yielding constant GFP expression (GFP), (**A**) at 4 hrs and (**B**) at 24 hrs after bacterial colonization of LEMs. LEMs were incubated for 4 or 24 hrs with the different bacterial strains, subsequently fixed in 1% paraformaldehyde, and stained with DAPI. *hla* expressing bacteria are presented in green, DAPI staining is presented in blue. Scale bars = 50 μm.

The differential detection of alpha-toxin, an important virulence factor during skin infections caused by *S*. *aureus*, in biofilms of different strains on LEMs and PS was further investigated using GFP-reporter technology. Visualization of alpha-toxin produced by *S*. *aureus* was performed by using strains LUH14616 and Sac042w transformed with a vector containing the promoter for *hla*, coupled to GFP. Using fluorescence microscopy, small microcolonies of these bacteria were observed that did not express *hla* after 4 hrs of colonization of epidermal models (Fig 5A), whereas at 24 hrs of colonization LUH14616 highly expressed *hla*, as indicated by the green fluorescent signal (Fig 5B). In contrast to results seen at the protein level, *hla* expression by LUH14616 was also visualized after 24 hrs of colonization of polystyrene (S2 Fig), suggesting that the gene is transcribed but not translated and/or that protein is rapidly degraded. Similar results were obtained for Sac042w, although this was less pronounced than for LUH14616 (Fig 5B, S2 Fig).

## Discussion

In this study we established the presence of 52 proteins in biofilms of five genetically different *S*. *aureus* strains on two different types of surfaces, i.e. Leiden epidermal modes (LEMs) and polystyrene (PS). We detected six functionally diverse proteins in biofilms of three different strains on both surfaces. Several of these proteins, including ClfB, glucosaminidase and SA0688, have been previously associated with biofilm formation [39–42], although so far not on a human biotic surface. In this context, surface- and strain-dependent differences in the presence of a wide range of proteins, including alpha-toxin, were found. The detection of multiple toxins (HlgB, LukD/E and alpha toxin) in biofilms of multiple strains on LEMs, but not PS, indicates surface specific protein expression. This implicates that currently used biofilm models, such as those on PS, might not adequately reflect biofilm formation on a more complex surface, such as the human skin. However, we should realize that the biofilms on LEMs and PS were formed under different conditions, i.e. submerged in culture medium for biofilms on PS and on an air-exposed, dry surface in case of biofilm formation on LEMs.

Biofilm formation by strain LUH14616 on LEMs and PS was confirmed using EM. Interestingly, bacteria in a biofilm on LEM but not on PS were completely encased by an extracellular matrix, indicating a phenotypic difference in bacterial biofilm formation on the two models. However, in the current study we did not characterize the material encasing bacteria in more detail, e.g. using immunoelectron microscopy [43].

The detection of *S*. *aureus* toxins, most notably alpha-toxin, in biofilms on LEMs is in agreement with their well-established roles in the pathogenesis of skin infections [18,44]. The cytolytic pore-forming alpha-toxin [45] lyses human cells including skin tissue, interferes with the innate and adaptive immune responses in a murine skin infection model [46], and is essential for biofilm development on mucosal surfaces [47]. Interestingly, in human skin, the filaggrin protein may inhibit alpha-toxin’s cytotoxicity by its ability to regulate the secretion of sphingomyelinase [48]. In line with this, >90% of the atopic dermatitis (AD) patients, who often have reduced filaggrin expression, are colonized by *S*. *aureus* [49], whereas about 25% of the normal population is persistently colonized by this bacterium [50]. Moreover, *S*. *aureus* strains isolated from AD patients displayed a higher alpha-toxin production than strains from healthy controls, while the amount of alpha-toxin produced was correlated with disease severity [51].

The other toxins detected in biofilms in this study, including HlgB and the leukocidins D/ E, have also been associated with *S*. *aureus* skin colonization and infection. This is supported by data from both murine models [52,53] and clinical-epidemiological studies [54,55]. Other data also supports the presence of other, non-toxin proteins detected in this study. For instance, the detected lipase might support the persistence of *S*. *aureus* in the fatty secretions of mammalian skin [56,57]. A recent study demonstrated that lipases are essential for *S*. *aureus* biofilm formation [56].

The PHG used in this study to establish bacterial protein presence consisted of a previously described pool of serum from both nasal and non-nasal carriers of *S*. *aureus* [35]. Specific IgG against all tested proteins was detected in PHG and these IgG levels were generally higher than in serum from individual patients suffering from a *S*. *aureus* bacteremia [27] (unpublished data). Combined with the high sensitivity of the Luminex assay [58,59] we think that it is unlikely that the current CLA would not detect antibody absorption by IgG-accessible proteins. However, future studies using other antibody sources (e.g. specific monoclonal antibodies) might further increase the sensitivity of this assay.

A limitation of the PHG used in this study is the aspecific decrease in IgG that was observed against leukocidins S and F, while genes for these proteins were not present in strain LUH14616 and proteomics data could not confirm the presence of HlgB or Luk D/E in 8-hrs biofilms on PS. The known immunological cross-reactivity between Luk D/E, S/F and HlgB [60,61] may explain these conflicting findings. In addition, an incomplete protein library used during mass-spec analysis might explain why CLA results for a particular protein could not be confirmed. Additional mass-spectrometry should be performed on mature biofilms on LEMs to confirm or exclude the presence of these proteins.

Expression on LEMs of *hla*, the gene encoding alpha toxin, was confirmed for two strains using GFP reporter technology. The low levels of *hla* expression by the USA300-derived strain Sac042w may be explained by strain-specific traits [62], possibly caused by mutations in upstream regulators such as *sarA* [63]. Interestingly, for strain LUH14616 *hla* expression was also observed on PS, while CLA nor mass-spectrometry detected alpha toxin at the protein level on this surface. This suggests that *hla* is transcribed but not translated and/or that the protein is rapidly degraded by the bacteria on PS, which has been observed previously for other *S*. *aureus* strains during planktonic growth [63–66].

Regulation of *hla* and other genes for *S*. *aureus* virulence factors is influenced by many factors, including the accessory gene regulatory locus (Agr), RNAIII [67], downstream transcription factors Rot [68,69], SarA and -S and Sae [70]. In this connection, we noted that several proteins detected during biofilm formation on LEMs, including alpha-toxin, LytM, SSLs and Spa are (indirectly) regulated by RNAIII [67,71]. Moreover, RNAIII can also directly interfere with mRNA of LytM leading to its down-regulation [72]. Therefore, it may well be that the quorum sensing system of AgrA/RNAIII of *S*. *aureus* is activated differently upon interaction of *S*. *aureus* with either LEMs or PS, leading to up- or down-regulation of specific genes depending on the surface. However, further studies including quantitative mass-spectrometry and transcriptomic analysis are necessary to clarify the role of the diverse regulatory systems [73–75] involved in the expression of *hla* and other genes during biofilm formation on LEMs.

In the context of new anti-infective therapies, such as vaccines, our data indicate that diverse proteins of *S*. *aureus* in biofilms are accessible to human IgG. Although biofilm-associated bacteria are thought to be more resistant to antimicrobials and effectors of the human immune system [13–15], our data suggests that, in addition to animal models [41], also vaccine-boosted human antibodies can target biofilms. Further insights into the functionality of antibodies, specifically in regard to (the inhibition of) biofilm growth, are required.

Alternatively, it may be interesting to choose an anti-virulence based therapy, for example by targeting interfering RNAs, such as RNAIII that affect the expression of many virulence factors [76]. For example, the RnpA- inhibitor RNPA1000, was shown to have *in vitro* antimicrobial effect against *S*. *aureus* (and other gram positive pathogens). Moreover, this enzyme dose-dependently protected against the pathogenesis of *S*. *aureus* in a mouse infection model [77]. Based on our data such an anti-virulence therapy may be effective against biofilms on skin of e.g. AD patients colonized by *S*. *aureus*, but not against biofilms on abiotic surfaces, such as that of a colonized catheter.

We conclude that functionally diverse virulence factors of (methicillin-resistant) *S*. *aureus* are present during biofilm formation on PS and LEMs. We specifically confirmed the presence of alpha-toxin during biofilm formation of MRSA strains LUH14616 and Sac042w on LEMs. In addition, the presence of several toxins, including alpha-toxin, immune modulators and other proteins appear to differ depending on the studied strain and surface. These observations merit more mechanistic studies to elucidate the function of specific proteins and the regulation of their expression within *S*. *aureus* biofilms. However, the present data further suggests that specific proteins, such as the ubiquitously present IsdA or SA0688, could be potential targets for novel agents to prevent biofilm formation and/or to reduce biofilm formation not only in animal models but also on human biotic surfaces.

## Supporting Information

S1 Fig Detection of *S*. *aureus* toxins and immune-modulators in 24 hrs biofilms of LUH14616 and surrounding medium. Results are shown for (**A**) alpha-toxin, (**B**) HlgB, (**C)** FlipR, and (**D**) SSL1. Remaining non-bound IgG specific against the different proteins was separately measured after incubation of PHG with biofilms on PS and after incubation with the IMDM culture medium covering the biofilms. Closed squares indicate IgG measurements from the biofilm samples and open triangles indicate measurements from medium samples. Biofilm mass on PS is indicated by open squares, which are plotted on the right Y-axis. Dashed horizontal lines indicate average MFI of sterile controls. Results are presented as the mean of 2–4 experiments.
(TIF)

S2 Fig Expression of *hla* by *S*. *aureus* LUH14616 and Sac042w during biofilm formation on PS. LUH14616 and Sac042w containing *hla*-GFP (*hla)*, an empty vector or a construct yielding constant GFP expression (GFP), at 4 hrs and 24 hrs after bacterial colonization of PS. *hla* expressing bacteria are presented in green.
(TIF)

S1 File Bacterial proteins used for the competitive Luminex assay. (DOCX)

S1 Table Detection of mRNA and proteins in biofilms of three *S*. *aureus* strains on LEMs and PS. (DOCX)
