# Correction: Detection of Alpha-Toxin and Other Virulence Factors in Biofilms of *Staphylococcus aureus* on Polystyrene and a Human Epidermal Model

**DOI:** 10.1371/journal.pone.0152544

**Published:** 2016-03-24

**Authors:** P. M. den Reijer, E. M. Haisma, N. A. Lemmens-den Toom, J. Willemse, R. I. Koning, J. A. A. Demmers, D. H. W. Dekkers, E. Rijkers, A. El Ghalbzouri, P. H. Nibbering, W. van Wamel

The fifth author’s name is incorrectly spelled. The correct name is: R.I. Koning. The correct citation is: den Reijer PM, Haisma EM, Lemmens-den Toom NA, Willemse J, Koning RI, Demmers JAA, et al. (2016) Detection of Alpha-Toxin and Other Virulence Factors in Biofilms of *Staphylococcus aureus* on Polystyrene and a Human Epidermal Model. PLoS ONE 11(1): e0145722. doi:10.1371/journal.pone.0145722
