# Supplementary figures and images for: Detection of Alpha-Toxin and Other Virulence Factors in Biofilms of Staphylococcus aureus on Polystyrene and a Human Epidermal Model
Source: PLoS One. 2016 Jan 7;11(1):e0145722. doi: 10.1371/journal.pone.0145722 (PMC4704740; doi:10.1371/journal.pone.0145722)

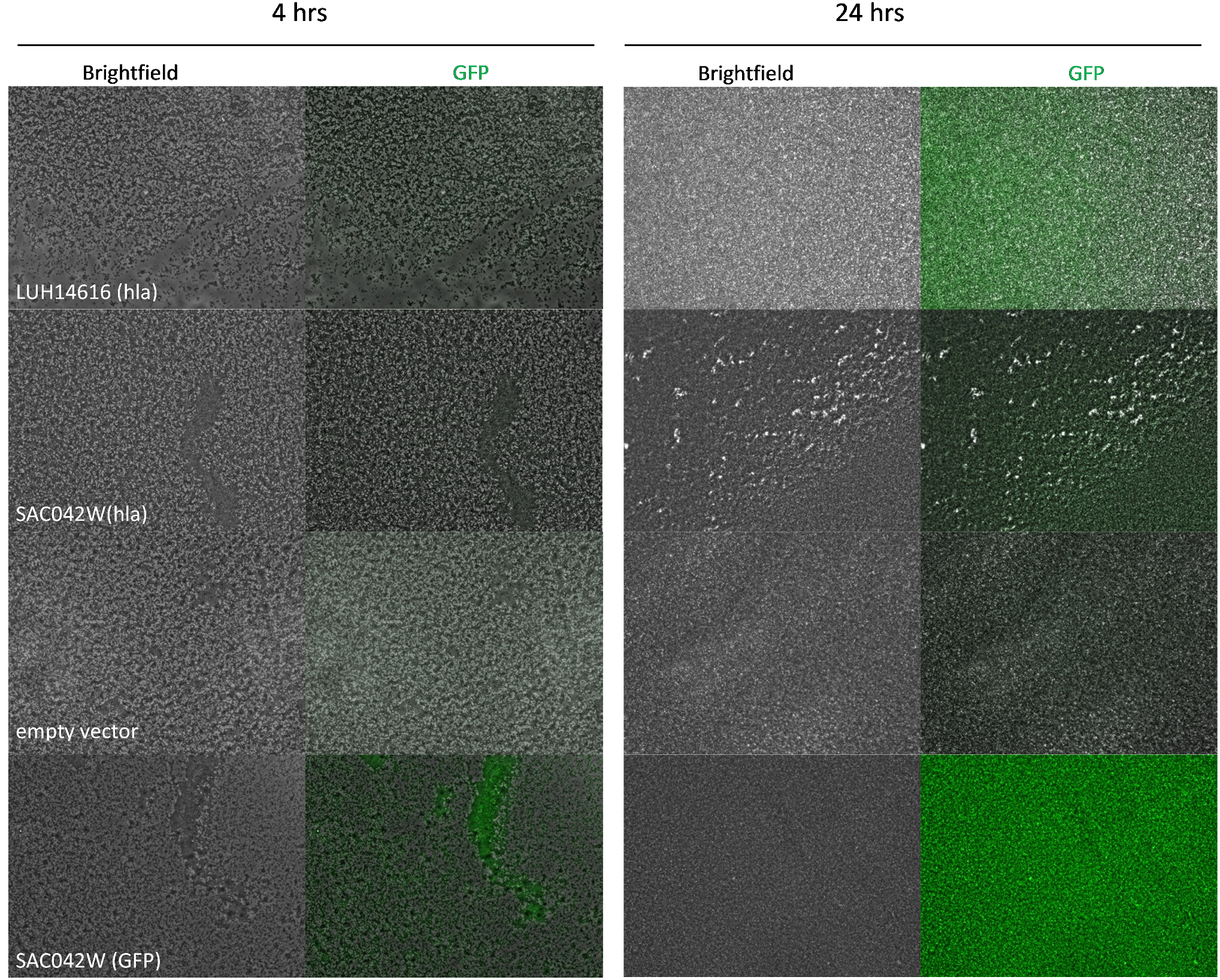

Supplement: S2 Fig — LUH14616 and Sac042w containing hla-GFP (hla), an empty vector or a construct yielding constant GFP expression (GFP), at 4 hrs and 24 hrs after bacterial colonization of PS. hla expressing bacteria are presented in green. (TIF) [file pone.0145722.s002.tif]
