# Supplementary material for: Detection of Alpha-Toxin and Other Virulence Factors in Biofilms of Staphylococcus aureus on Polystyrene and a Human Epidermal Model
Source: PLoS One. 2016 Jan 7;11(1):e0145722. doi: 10.1371/journal.pone.0145722 (PMC4704740; doi:10.1371/journal.pone.0145722)
Supplement: S1 Table — (DOCX) [file pone.0145722.s004.docx]

|  |  | Sterile control 24 hrs | |  | Luh14616 24 hrs biofilms | |  |  | Luh15051 24 hrs biofilms | |  |  | Saco042 (USA300) 24 hrs biofilms | | |
| --- | --- | --- | --- | --- | --- | --- | --- | --- | --- | --- | --- | --- | --- | --- | --- |
|  |  | Non-bound IgG in MFI ± SD | |  | Non-bound IgG in MFI ± SD, (% reduction)^1^ | | |  | Non-bound IgG in MFI ± SD, (% reduction)^1^ | | |  | Non-bound IgG in MFI ± SD, (% reduction)^1^ | | |
| Protein | Functional class | Polystyrene | LEMs^2^ |  | Polystyrene | LEMs^2^ | Gene present^3^ |  | Polystyrene | LEMs^2^ | Gene present^3^ |  | Polystyrene | LEMs^2^ | Gene present^3^ |
| Alpha toxin | toxin | 5033 ± 768 | 4957 ± 77 |  | 4896 ± 860 (3) | 294 ± 36 (94)* | yes |  | 4128 ± 89 (18) | 185 ± 27 (96)* | yes |  | 2705 ± 323 (46)* | 151 ± 68 (97)* | yes |
| CHIPS | immmune modulator | 6917 ± 1277 | 6508 ± 87 |  | 4172 ± 2200 (40)* | 1020 ± 729 (85)* | yes |  | 2817 ± 554 (59)* | 1274 ± 988 (80)* | yes |  | 746 ± 15 (89)* | 1279 ± 675 (80)* | yes |
| ClfA | surface protein | 768 ± 209 | 767 ± 28 |  | 585 ± 78 (24) | 660 ± 22 (14) | no |  | 423 ± 74 (45)* | 105 ± 9 (86)* | yes |  | 273 ± 2 (64)* | 172 ± 124 (78)* | yes |
| ClfB | surface protein | 485 ± 237 | 356 ± 128 |  | 187 ± 87 (61)* | 72 ± 30 (80)* | yes |  | 249 ± 73 (49) | 41 ± 10 (88)* | yes |  | 179 ± 12 (63)* | 92 ± 43 (74)* | yes |
| Efb | immmune modulator | 2706 ± 802 | 2541 ± 176 |  | 1031 ± 101 (62)* | 1094 ± 158 (57)* | yes |  | 485 ± 68 (82)* | 502 ± 96 (80)* | yes |  | 967 ± 502 (64)* | 1087 ± 883 (57)* | yes |
| EsxA | housekeeping | ND^4^ |  |  |  |  | yes |  |  |  | yes |  |  |  | yes |
| EsxB | housekeeping | ND^4^ |  |  |  |  | yes |  |  |  | no |  |  |  | yes |
| ETA | toxin | 192 ± 23 | 196 ± 18 |  | 201 ± 11 (0) | 257 ± 13 (0) | no |  | 184 ± 6 (4) | 81 ± 18 (59)* | no |  | 179 ± 21 (7) | 135 ± 9 (31) | no |
| ETB | toxin | 50 ± 12 | 49 ± 4 |  | 78 ± 11 (0) | 121 ± 42 (0) | no |  | 55 ± 2 (0) | 45 ± 7 (8) | no |  | 103 ± 46 (0) | 47 ± 8 (4) | no |
| FlipR | immmune modulator | 1211 ± 294 | 1088 ± 317 |  | 950 ± 34 (22) | 322 ± 138 (70)* | yes |  | 890 ± 25 (26) | 90 ± 51 (92)* | yes |  | 632 ± 81 (46)* | 584 ± 231 (47)* | yes |
| FnbA | surface protein | 461 ± 128 | 399 ± 17 |  | 383 ± 87 (17) | 257 ± 79 (36) | yes |  | 265 ± 51 (43)* | 146 ± 22 (63)* | yes |  | 282 ± 19 (39)* | 227 ± 107 (43)* | yes |
| FnbB | surface protein | 82 ± 36 | 59 ± 12 |  | 39 ± 5 (52)* | 31 ± 15 (48)* | yes |  | 36 ± 5 (56)* | 36 ± 5 (39) | yes |  | 52 ± 10 (37)* | 47 ± 17 (20) | yes |
| Glucosaminidase | housekeeping | 2088 ± 736 | 1699 ± 563 |  | 425 ± 190 (80) | 140 ± 4 (92)* | yes |  | 1200 ± 39 (43)* | 40 ± 8 (98)* | yes |  | 241 ± 26 (88)* | 81 ± 7 (95)* | yes |
| HlgB | toxin | 5145 ± 2612 | 5800 ± 102 |  | 5029 ± 824 (3) | 465 ± 283 (92)* | yes |  | 3682 ± 1062 (28) | 316 ± 263 (95)* | yes |  | 3135 ± 223 (39)* | 663 ± 444 (89)* | yes |
| IsaA | housekeeping | 3028 ± 1178 | 2516 ± 773 |  | 379 ± 27 (87)* | 293 ± 29 (88)* | yes |  | 370 ± 50 (88)* | 92 ± 8 (96)* | yes |  | 469 ± 123 (84)* | 385 ± 89 (85)* | yes |
| IsdA | surface protein | 1526 ± 704 | 1628 ± 60 |  | 178 ± 62 (88)* | 262 ± 89 (84)* | yes |  | 83 ± 17 (95)* | 154 ± 54 (90)* | yes |  | 151 ± 85 (90)* | 179 ± 74 (89)* | yes |
| IsdH | surface protein | ND^4^ |  |  |  |  | yes |  |  |  | yes |  |  |  | yes |
| Lipase | housekeeping/ toxin | 1598 ± 423 | 1534 ± 49 |  | 1336 ± 125 (16) | 444 ± 60 (71)* | yes |  | 662 ± 317 (59)* | 70 ± 33 (95)* | yes |  | 250 ± 128 (84)* | 41 ± 25 (97)* | yes |
| LukD | toxin | 4950 ± 1134 | 4580 ± 8 |  | 4481 ± 598 (9) | 1716 ± 362 (62)* | yes |  | 3857 ± 74 (22) | 1020 ± 498 (78)* | yes |  | 3136 ± 237 (37)* | 1601 ± 1125 (65)* | yes |
| LukE | toxin | 4607 ± 1297 | 4434 ± 87 |  | 4143 ± 474 (10) | 1538 ± 191 (65)* | yes |  | 3588 ± 53 (22) | 1208 ± 318 (73)* | yes |  | 3164 ± 190 (31) | 1807 ± 952 (59)* | yes |
| LukF | toxin | 672 ± 219 | 524 ± 204 |  | 674 ± 173 (0) | 361 ± 41 (31) | no |  | 478 ± 11 (29) | 87 ± 9 (83)* | yes |  | 408 ± 78 (39)* | 92 ± 18 (82)* | yes |
| LukS | toxin | 2699 ± 864 | 2644 ± 127 |  | 2338 ± 652 (13) | 335 ± 82 (87)* | no |  | 1878 ± 56 (30) | 326 ± 218 (88)* | yes |  | 1352 ± 92 (50)* | 206 ± 174 (92)* | yes |
| LytM | housekeeping | 325 ± 73 | 312 ± 14 |  | 223 ± 4 (31) | 60 ± 16 (80)* | yes |  | 199 ± 23 (39)* | 53 ± 2 (83)* | yes |  | 197 ± 37 (39)* | 82 ± 62 (74)* | yes |
| Nuc | housekeeping/ toxin | 872 ± 198 | 704 ± 254 |  | 401 ± 100 (50)* | 442 ± 58 (37)* | yes |  | 208 ± 55 (76)* | 90 ± 12 (87)* | yes |  | 299 ± 214 (66)* | 156 ± 31 (78)* | yes |
| PrsA | housekeeping | ND^4^ |  |  |  |  | yes |  |  |  | yes |  |  |  | yes |
| SACOL0486 | housekeeping | 234 ± 203 | 514 ± 12 |  | 208 ± 99 (11) | 540 ± 227 (0) | no |  | 71 ± 1 (70)* | 231 ± 126 (55)* | no |  | 288 ± 276 (0) | 139 ± 92 (73)* | no |
| SACOL0688 | housekeeping | 297 ± 72 | 277 ± 91 |  | 82 ± 5 (72)* | 97 ± 16 (65)* | yes |  | 141 ± 42 (53)* | 35 ± 6 (87)* | yes |  | 112 ± 49 (62)* | 67 ± 33 (75)* | yes |
| SasG | surface protein | 173 ± 49 | 144 ± 18 |  | 161 ± 24 (7) | 154 ± 32 (0) | yes |  | 100 ± 15 (42)* | 52 ± 7 (64)* | yes |  | 106 ± 37 (39)* | 53 ± 20 (63)* | yes |
| SCIN | immmune modulator | 3178 ± 411 | 4013 ± 196 |  | 2977 ± 706 (6) | 3625 ± 306 (10) | yes |  | 298 ± 81 (91)* | 223 ± 63 (94)* | yes |  | 365 ± 69 (89)* | 1804 ± 1139 (55)* | yes |
| SdrD | surface protein | 85 ± 38 | 83 ± 4 |  | 70 ± 17 (18) | 52 ± 8 (38) | yes |  | 56 ± 2 (34) | 53 ± 5 (36) | yes |  | 51 ± 2 (40)* | 64 ± 22 (23) | yes |
| SdrE | surface protein | 299 ± 75 | 299 ± 50 |  | 407 ± 117 (0) | 497 ± 18 (0) | no |  | 247 ± 21 (17) | 95 ± 26 (69)* | yes |  | 317 ± 108 (0) | 135 ± 2 (55)* | yes |
| SEA | toxin | 666 ± 221 | 585 ± 49 |  | 463 ± 27 (30) | 187 ± 17 (68)* | yes |  | 435 ± 20 (35)* | 290 ± 13 (50)* | no |  | 370 ± 43 (45)* | 376 ± 120 (36) | no |
| SEB | toxin | 818 ± 222 | 751 ± 50 |  | 688 ± 107 (16) | 675 ± 43 (10) | no |  | 587 ± 30 (28) | 378 ± 31 (50)* | no |  | 533 ± 73 (35)* | 514 ± 174 (32) | no |
| SEC | toxin | 4476 ± 1094 | 4241 ± 175 |  | 3702 ± 603 (17) | 3759 ± 174 (12) | no |  | 3260 ± 104 (27) | 2176 ± 128 (49) | no |  | 2677 ± 268 (40)* | 3011 ± 960 (29) | no |
| SED | toxin | 157 ± 45 | 147 ± 7 |  | 131 ± 12 (17) | 106 ± 11 (18) | no |  | 114 ± 1 (18) | 65 ± 2 (65)* | no |  | 94 ± 8 (40)* | 86 ± 48 (42)* | no |
| SEE | toxin | 79 ± 21 | 75 ± 8 |  | 53 ± 2 (33) | 25 ± 8 (67)* | no |  | 57 ± 7 (28) | 40 ± 1 (47)* | no |  | 59 ± 13 (25) | 53 ± 16 (29) | no |
| SEG | toxin | 87 ± 15 | 85 ± 3 |  | 190 ± 43 (0) | 194 ± 18 (0) | no |  | 98 ± 14 (0) | 91 ± 6 (0) | no |  | 209 ± 81 (0) | 88 ± 22 (0) | no |
| SEH | toxin | 289 ± 69 | 300 ± 25 |  | 275 ± 34 (5) | 318 ± 17 (0) | no |  | 237 ± 23 (18) | 169 ± 11 (44)* | no |  | 261 ± 100 (10) | 213 ± 84 (29) | no |
| SEI | toxin | 108 ± 38 | 97 ± 5 |  | 136 ± 26 (0) | 157 ± 37 (0) | no |  | 101 ± 18 (6) | 81 ± 20 (17) | no |  | 165 ± 125 (0) | 71 ± 5 (27) | no |
| SEJ | toxin | ND^4^ |  |  |  |  | no |  |  |  | no |  |  |  | no |
| SEM | toxin | 107 ± 43 | 95 ± 7 |  | 68 ± 2 (37)* | 69 ± 6 (27) | no |  | 68 ± 11 (37)* | 45 ± 1 (47)* | no |  | 60 ± 20 (44)* | 48 ± 18 (50)* | no |
| SEN | toxin | 73 ± 5 | 91 ± 5 |  | 195 ± 55 (0) | 278 ± 14 (0) | no |  | 75 ± 8 (0) | 58 ± 7 (36) | no |  | 165 ± 158 (0) | 109 ± 22 (0) | no |
| SEO | toxin | 47 ± 14 | 46 ± 6 |  | 36 ± 1 (24) | 57 ± 20 (0) | no |  | 36 ± 2 (23) | 24 ± 1 (48)* | no |  | 34 ± 9 (28) | 32 ± 8 (30) | no |
| SEQ | toxin | 98 ± 31 | 94 ± 9 |  | 71 ± 15 (28) | 62 ± 3 (34) | no |  | 66 ± 5 (33) | 39 ± 1 (59)* | no |  | 45 ± 3 (54)* | 40 ± 16 (58)* | yes |
| SER | toxin | 94 ± 10 | 118 ± 19 |  | 121 ± 17 (0) | 152 ± 8 (0) | no |  | 90 ± 2 (4) | 49 ± 15 (59)* | no |  | 92 ± 9 (2) | 85 ± 14 (28) | no |
| SSL1 | immmune modulator | 1754 ± 474 | 1719 ± 48 |  | 1441 ± 25 (18) | 422 ± 236 (75)* | yes |  | 1261 ± 113 (28) | 274 ± 36 (84)* | no |  | 1071 ± 61 (39)* | 938 ± 620 (35) | yes |
| SSL3 | immmune modulator | 2902 ± 816 | 2782 ± 71 |  | 2412 ± 364 (17) | 2470 ± 69 (11) | yes |  | 2102 ± 79 (28) | 1559 ± 68 (44)* | no |  | 1795 ± 223 (39)* | 1999 ± 485 (29) | yes |
| SSL5 | immmune modulator | 1081 ± 317 | 1007 ± 70 |  | 907 ± 141 (16) | 1006 ± 143 (0) | yes |  | 756 ± 41 (30) | 614 ± 34 (39) | no |  | 786 ± 208 (28) | 763 ± 147 (24) | yes |
| SSL9 | immmune modulator | 2700 744 | 2379 ± 109 |  | 2596 ± 631 (4) | 977 ± 66 (59)* | no |  | 2027 ± 60 (25) | 1331 ± 14 (44)* | no |  | 2058 ± 540 (24) | 1874 ± 435 (21) | no |
| SSL10 | immmune modulator | 2434 ± 825 | 2224 ± 299 |  | 2073 ± 336 (15) | 1906 ± 342 (14) | yes |  | 1815 ± 3 (25) | 868 ± 260 (61)* | yes |  | 1573 ± 266 (35)* | 1611 ± 185 (28) | yes |
| SSL11 | immmune modulator | 335 ± 96 | 350 ± 41 |  | 284 ± 18 (15) | 280 ± 12 (20) | no |  | 258 ± 3 (23) | 105 ± 39 (70)* | no |  | 228 ± 40 (32) | 246 ± 48 (30) | no |
| TSST1 | toxin | 4208 ± 1087 | 3999 ± 168 |  | 3506 ± 650 (17) | 3598 ± 176 (10) | no |  | 3104 ± 86 (26) | 2270 ± 177 (43)* | no |  | 2600 ± 278 (38)* | 2944 ± 694 (26) | no |

**S1 table. Detection of mRNA and proteins in biofilms of three *S. aureus* strains on LEMs and PS.**

^1^Percentage decrease of specific IgG for each protein was calculated in relation to the negative control and can be considered as a semi-quantitative measure of protein-specific antibody absorption, indirectly reflecting the presence of the protein in the biofilm.

^2^Leiden human Epidermal Models

^3^Presence of genes was established using PCR.

^4^Not Determined, data were excluded due to low MFI’s with standard deviations larger than 25% between repeated CLA measurements.

^*^Reductions in specific IgG surpassing the cut-off values (35% antibody absorption at 24 hrs biofilm growth and 40% at 48 hrs), indicative of protein presence.
